# Supplementary material for: Taxon-specific redox conditions control fossilisation pathways
Source: Nat Commun. 2025 Apr 29;16:3993. doi: 10.1038/s41467-025-59372-3 (PMC12038014; doi:10.1038/s41467-025-59372-3)
Supplement: Supplementary file 1 — Supplementary Information [file 41467_2025_59372_MOESM1_ESM.pdf]

Taxon-specific redox conditions control fossilisation pathways

Nora Corthésy[1*], Jonathan B. Antcliffe[1], and Farid Saleh[1*]

[1]Institute of Earth Sciences, University of Lausanne, Géopolis, CH-1015 Lausanne, Switzerland

Corresponding authors: N. Corthésy (nora.corthesy@unil.ch)
F. Saleh (farid.nassim.saleh@gmail.com)

ORCID: N. Corthésy (0009-0005-4342-4641)
F. Saleh (0000-0002-9165-985X)

SUPPLEMENTARY MATERIALS

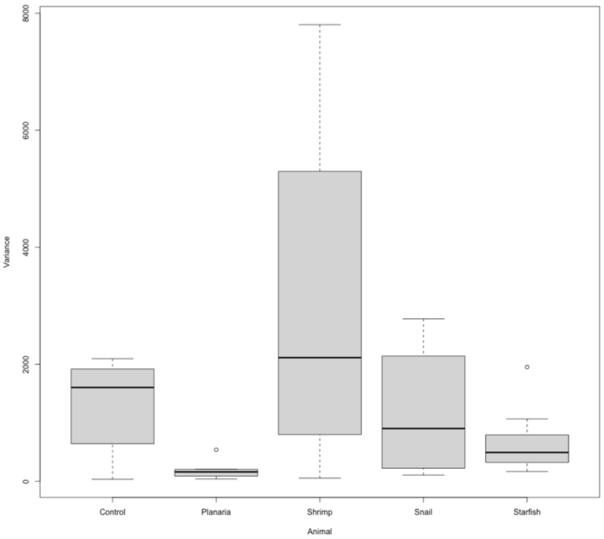

Figure S1. Variances across the raw ORP values of the different animals: control (n = 8), planaria (n = 8), shrimp (n = 8), snail (n = 20), starfish (n = 8).

Table S1. Two-sample t-test (two-sided) to compare variances across the raw ORP values between the different animals. Significant p-values mean that variances are different between two groups of animals and are highlighted in bold.

	Degree of freedom	*t-ratio*	*p-value*
**Snail and Shrimp**	9.009	1.747	0.115
**Snail and Starfish**	10.8	1.179	0.263
**Snail and Planarian**	7.297	-2.581	**0.035**
**Snail and Control**	7.605	2.335	**0.049**
**Shrimp and Starfish**	7.605	2.335	**0.049**
**Shrimp and Planarian**	7.044	-2.865	**0.024**
**Shrimp and Control**	7.588	4.071	**0.004**
**Starfish and Planarians**	13.1	1.885	0.082
**Starfish and Control**	13.1	1.885	0.082
**Planarian and Control**	8.003	-2.253	0.054

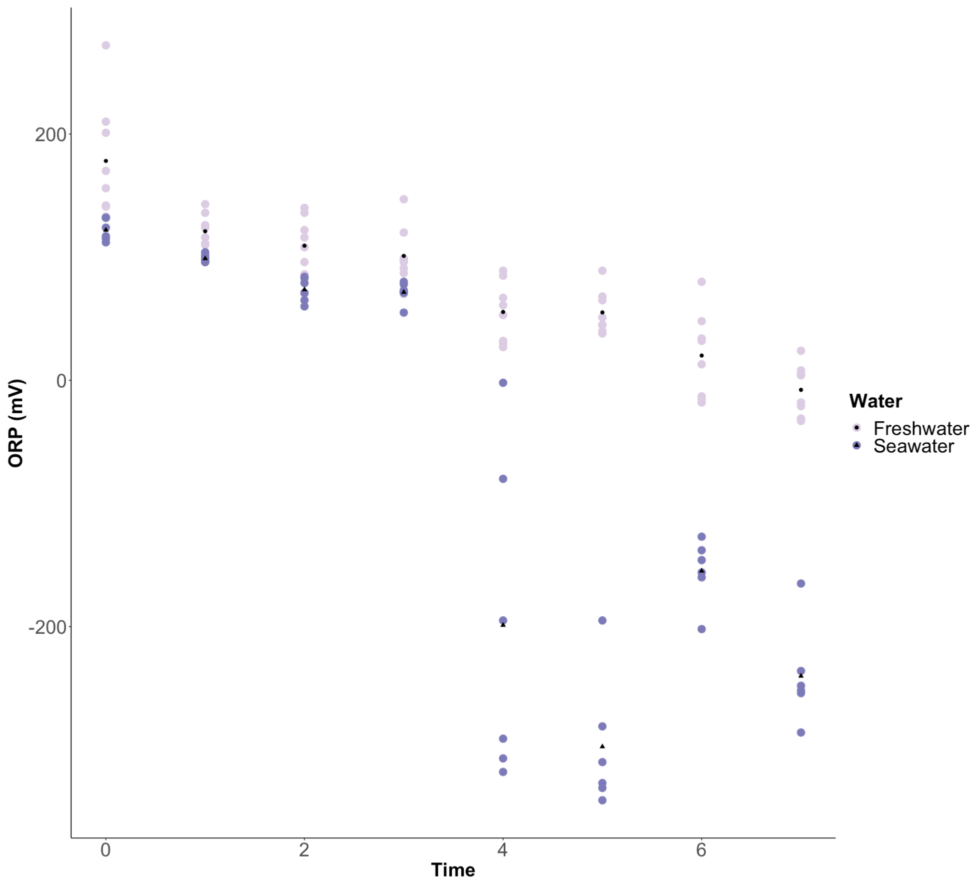

Figure S2. Oxidation-reduction potential (ORP) values (mV) over time for starfish decaying in freshwater and saltwater. ORP values reflect different redox zonations: nitrification (+100 to +350 mV), carbonaceous biochemical oxygen demand (cBOD) (+50 to +250 mV), denitrification (-50 to +50 mV), sulphate reduction (-250 to -50 mV), biological phosphorus release (-250 to -100 mV), and methanogenesis (-400 to -175 mV). The average values of ORP of starfish decaying in freshwater (n = 8) and in seawater (n = 8) at each time point are represented by a black dot and a black triangle respectively.

Table S2. Contrast analysis of generalized linear model (two-sided) to compare the ORP values between the animals (planarians, shrimp, snail, starfish, control without animals) at each timepoint (0-7 days) of the experiment. Significant p-values are highlighted in bold. The higher the t-ratio, the greater the chance that the null hypothesis will be rejected.

	Estimate	*Standard Error*	*t-ratio*	*p-value*
**Time = 0 day**				
Control – Planarians	-48.14	17.8	-2.704	0.0553
Control – Shrimp	-50.77	17.8	-2.852	**0.0369**
Control – Snail	-91.62	15.2	-6.024	**< 0.0001**
Control – Starfish	-21.27	17.8	-1.195	0.7545
Planarians – Shrimp	-2.62	17.2	-0.153	0.9999
Planarians – Snail	-43.47	14.5	-2.999	0.0241
Planarians – Starfish	26.88	17.2	1.562	0.5225
Shrimp – Snail	-40.85	14.5	-2.817	0.0406
Shrimp – Starfish	29.50	17.2	1.715	0.4259
Snail – Starfish	70.35	14.5	4.852	**< 0.0001**
**Time = 1 day**				
Control – Planarians	-21.61	17.8	-1.214	0.7435
Control – Shrimp	75.77	17.8	4.256	**0.0003**
Control – Snail	-2.96	15.2	-0.195	0.9997
Control – Starfish	49.14	17.8	2.760	**0.0475**
Planarians – Shrimp	97.38	17.2	5.661	**< 0.0001**

Planarians – Snail	18.64	14.5	1.286	0.7000
Planarians – Starfish	70.75	17.2	4.113	**0.0005**
Shrimp – Snail	-78.73	14.5	-5.430	**< 0.0001**
Shrimp – Starfish	-26.62	17.2	-1.548	0.5320
Snail – Starfish	52.11	14.5	3.594	**0.0034**
**Time = 2 days**				
Control – Planarians	-16.14	17.8	-0.907	0.8942
Control – Shrimp	209.73	17.8	11.780	**< 0.0001**
Control – Snail	163.70	15.2	10.763	**< 0.0001**
Control – Starfish	62.61	17.8	3.517	**0.0045**
Planarians – Shrimp	225.88	17.2	13.132	**< 0.0001**
Planarians – Snail	179.84	14.5	12.404	**< 0.0001**
Planarians – Starfish	78.75	17.2	4.578	**0.0001**
Shrimp – Snail	-46.03	14.5	-3.175	**0.0140**
Shrimp – Starfish	-147.12	17.2	-8.554	**< 0.0001**
Snail – Starfish	-101.09	14.5	-6.973	**< 0.0001**
**Time = 3 days**				
Control – Planarians	-8.70	17.8	-0.488	0.9884
Control – Shrimp	290.80	17.8	16.334	**< 0.0001**
Control – Snail	213.95	15.2	14.067	**< 0.0001**
Control – Starfish	71.43	17.8	4.012	**0.0007**
Planarians – Shrimp	299.50	17.2	17.413	**< 0.0001**
Planarians – Snail	222.65	14.5	15.357	**< 0.0001**
Planarians – Starfish	80.12	17.2	4.658	**< 0.0001**
Shrimp – Snail	-76.85	14.5	-5.300	**< 0.0001**
Shrimp – Starfish	-219.38	17.2	-12.754	**< 0.0001**
Snail – Starfish	-142.53	14.5	-9.830	**< 0.0001**
**Time = 4 days**				
Control – Planarians	-15.93	17.8	-0.895	0.8988
Control – Shrimp	309.95	17.8	17.409	**< 0.0001**
Control – Snail	215.52	15.2	14.170	**< 0.0001**
Control – Starfish	111.07	17.8	6.239	**< 0.0001**
Planarians – Shrimp	325.88	17.2	18.946	**< 0.0001**
Planarians – Snail	231.45	14.5	15.964	**< 0.0001**
Planarians – Starfish	127.00	17.2	7.384	**< 0.0001**
Shrimp – Snail	-94.43	14.5	-6.513	**< 0.0001**
Shrimp – Starfish	-198.88	17.2	-11.562	**< 0.0001**
Snail – Starfish	-104.45	14.5	-7.204	**< 0.0001**
**Time = 5 days**				
Control – Planarians	-11.57	17.8	-0.650	0.8988
Control – Shrimp	364.43	17.8	20.469	**< 0.0001**
Control – Snail	214.17	15.2	14.081	**< 0.0001**
Control – Starfish	110.30	17.8	6.196	**< 0.0001**
Planarians – Shrimp	376.00	17.2	21.860	**< 0.0001**
Planarians – Snail	225.74	14.5	15.570	**< 0.0001**
Planarians – Starfish	121.88	17.2	7.086	**< 0.0001**

Shrimp – Snail	-150.26	14.5	-10.364	**< 0.0001**
Shrimp – Starfish	-254.12	17.2	-14.775	**< 0.0001**
Snail – Starfish	-103.86	14.5	-7.164	**< 0.0001**
**Time = 6 days**				
Control – Planarians	4.64	17.8	0.261	0.9990
Control – Shrimp	254.89	17.8	14.317	**< 0.0001**
Control – Snail	178.93	15.2	11.764	**< 0.0001**
Control – Starfish	119.02	17.8	6.685	**< 0.0001**
Planarians – Shrimp	250.25	17.2	14.549	**< 0.0001**
Planarians – Snail	174.29	14.5	12.021	**< 0.0001**
Planarians – Starfish	114.38	17.2	6.650	**< 0.0001**
Shrimp – Snail	-75.96	14.5	-5.239	**< 0.0001**
Shrimp – Starfish	-135.88	17.2	-7.900	**< 0.0001**
Snail – Starfish	-59.91	14.5	-4.132	**0.0004**
**Time = 7 days**				
Control – Planarians	-27.57	17.8	-1.549	0.5315
Control – Shrimp	219.68	17.8	12.339	**< 0.0001**
Control – Snail	167.32	15.2	11.001	**< 0.0001**
Control – Starfish	155.18	17.8	8.716	**< 0.0001**
Planarians – Shrimp	247.25	17.2	14.375	**< 0.0001**
Planarians – Snail	194.89	14.5	13.442	**< 0.0001**
Planarians – Starfish	182.75	17.2	10.625	**< 0.0001**
Shrimp – Snail	-52.36	14.5	-3.611	**0.0032**
Shrimp – Starfish	-64.50	17.2	-3.750	**0.0019**
Snail – Starfish	-12.14	14.5	-0.838	0.9187

Table S3. Contrast analysis of generalized linear model (two-sided) to compare the normalized ORP values (ORP values divided by the ORP values on day 0) between the animals (planarians, shrimp, snail, starfish, control without animals) at each timepoint (0-7 days) of the experiment. Significant p-values are highlighted in bold. The higher the t-ratio, the greater the chance that the null hypothesis will be rejected.

	Estimate	*Standard Error*	*t-ratio*	*p-value*
**Time = 0 day**				
Control – Planarians	0.0000	0.0862	0.0000	1.0000
Control – Shrimp	0.0000	0.0862	0.0000	1.0000
Control – Snail	0.0000	0.0737	0.0000	1.0000
Control – Starfish	0.0000	0.0862	0.0000	1.0000
Planarians – Shrimp	0.0000	0.0833	0.0000	1.0000
Planarians – Snail	0.0000	0.0702	0.0000	1.0000
Planarians – Starfish	0.0000	0.0833	0.0000	1.0000
Shrimp – Snail	0.0000	0.0702	0.0000	1.0000
Shrimp – Starfish	0.0000	0.0833	0.0000	1.0000
Snail – Starfish	0.0000	0.0702	0.0000	1.0000
**Time = 1 day**				
Control – Planarians	0.1502	0.0862	1.741	0.4100
Control – Shrimp	0.6297	0.0862	7.302	**< 0.0001**
Control – Snail	0.3880	0.0737	5.267	**< 0.0001**
Control – Starfish	0.3597	0.0862	4.171	**0.0004**
Planarians – Shrimp	0.4796	0.0833	5.756	**< 0.0001**

Planarians – Snail	0.2379	0.0702	3.387	**0.0070**
Planarians – Starfish	0.2095	0.0833	2.515	0.0896
Shrimp – Snail	-0.2417	0.0702	-3.442	**0.0058**
Shrimp – Starfish	-0.2700	0.0833	-3.241	**0.0113**
Snail – Starfish	-0.0283	0.0702	-0.404	0.9944
**Time = 2 days**				
Control – Planarians	0.1789	0.0862	2.075	0.2333
Control – Shrimp	1.2823	0.0862	14.869	**< 0.0001**
Control – Snail	1.0640	0.0737	14.442	**< 0.0001**
Control – Starfish	0.4324	0.0862	5.014	**< 0.0001**
Planarians – Shrimp	1.1034	0.0833	13.244	**< 0.0001**
Planarians – Snail	0.8851	0.0702	12.603	**< 0.0001**
Planarians – Starfish	0.2535	0.0833	3.043	**0.0211**
Shrimp – Snail	-0.2183	0.0702	-3.108	**0.0172**
Shrimp – Starfish	-0.8499	0.0833	-10.201	**< 0.0001**
Snail – Starfish	-0.6316	0.0702	-8.993	**< 0.0001**
**Time = 3 days**				
Control – Planarians	0.2158	0.0862	2.502	0.0.924
Control – Shrimp	1.6724	0.0862	19.393	**< 0.0001**
Control – Snail	1.2700	0.0737	17.238	**< 0.0001**
Control – Starfish	0.5075	0.0862	5.885	**< 0.0001**
Planarians – Shrimp	1.4566	0.0833	17.483	**< 0.0001**
Planarians – Snail	1.0542	0.0702	15.011	**< 0.0001**
Planarians – Starfish	0.2917	0.0833	3.502	**0.0047**
Shrimp – Snail	-0.4024	0.0702	-5.730	**< 0.0001**
Shrimp – Starfish	-1.1648	0.0833	-13.982	**< 0.0001**
Snail – Starfish	-0.7624	0.0702	-10.857	**< 0.0001**
**Time = 4 days**				
Control – Planarians	0.1725	0.0862	2.000	0.2680
Control – Shrimp	1.7556	0.0862	20.357	**< 0.0001**
Control – Snail	1.2621	0.0737	17.132	**< 0.0001**
Control – Starfish	0.7088	0.0862	8.219	**< 0.0001**
Planarians – Shrimp	1.5831	0.0833	19.001	**< 0.0001**
Planarians – Snail	1.0897	0.0702	15.516	**< 0.0001**
Planarians – Starfish	0.5363	0.0833	6.437	**< 0.0001**
Shrimp – Snail	-0.4934	0.0702	-7.026	**< 0.0001**
Shrimp – Starfish	-1.0468	0.0833	-12.564	**< 0.0001**
Snail – Starfish	-0.5534	0.0702	-7.879	**< 0.0001**
**Time = 5 days**				
Control – Planarians	0.1919	0.0862	2.225	0.1728
Control – Shrimp	2.0185	0.0862	23.407	**< 0.0001**
Control – Snail	1.2528	0.0737	17.005	**< 0.0001**
Control – Starfish	0.7131	0.0862	8.268	**< 0.0001**
Planarians – Shrimp	1.8267	0.0833	21.925	**< 0.0001**
Planarians – Snail	1.0609	0.0702	15.107	**< 0.0001**
Planarians – Starfish	0.5212	0.0833	6.255	**< 0.0001**

Shrimp – Snail	-0.7657	0.0702	-10.904	**< 0.0001**
Shrimp – Starfish	-1.3055	0.0833	-15.670	**< 0.0001**
Snail – Starfish	-0.5398	0.0702	-7.686	**< 0.0001**
**Time = 6 days**				
Control – Planarians	0.2317	0.0862	2.687	0.0579
Control – Shrimp	1.4544	0.0862	16.865	**< 0.0001**
Control – Snail	1.0480	0.0737	14.225	**< 0.0001**
Control – Starfish	0.7302	0.0862	8.467	**< 0.0001**
Planarians – Shrimp	1.2227	0.0833	14.676	**< 0.0001**
Planarians – Snail	0.8163	0.0702	11.623	**< 0.0001**
Planarians – Starfish	0.4985	0.0833	5.983	**< 0.0001**
Shrimp – Snail	-0.4064	0.0702	-5.787	**< 0.0001**
Shrimp – Starfish	-0.7242	0.0833	-8.693	**< 0.0001**
Snail – Starfish	-0.3178	0.0702	-4.525	**0.0001**
**Time = 7 days**				
Control – Planarians	0.0867	0.0862	1.006	0.8526
Control – Shrimp	1.2966	0.0862	15.035	**< 0.0001**
Control – Snail	1.0212	0.0737	13.862	**< 0.0001**
Control – Starfish	0.9705	0.0862	11.253	**< 0.0001**
Planarians – Shrimp	1.2099	0.0833	14.522	**< 0.0001**
Planarians – Snail	0.9345	0.0702	13.307	**< 0.0001**
Planarians – Starfish	0.8837	0.0833	10.607	**< 0.0001**
Shrimp – Snail	-0.2754	0.0702	-3.921	**0.0010**
Shrimp – Starfish	-0.3261	0.0833	-3.915	**0.0010**
Snail – Starfish	-0.0508	0.0702	-0.723	0.9511

Table S4. Contrast analysis of generalized linear model (two-sided) to compare the mass-corrected ORP values (normalized ORP values of animals subtracted from normalized ORP of controls, then divided by the average weight of animals) between the animals (planarians, shrimp, snail, starfish) at each timepoint (0-7 days) of the experiment. Significant p-values are highlighted in bold. The higher the t-ratio, the greater the chance that the null hypothesis will be rejected.

	Estimate	*Standard Error*	*t-ratio*	*p-value*
**Time = 0 day**				
Planarians – Shrimp	0.0000	1.177	0.0000	1.0000
Planarians – Snail	0.0000	0.992	0.0000	1.0000
Planarians – Starfish	0.0000	1.177	0.0000	1.0000
Shrimp – Snail	0.0000	0.992	0.0000	1.0000
Shrimp – Starfish	0.0000	1.177	0.0000	1.0000
Snail – Starfish	0.0000	0.992	0.0000	1.0000
**Time = 1 day**				
Planarians – Shrimp	9.380	1.177	7.972	**< 0.0001**
Planarians – Snail	7.033	0.992	7.091	**< 0.0001**
Planarians – Starfish	7.426	1.177	6.311	**< 0.0001**
Shrimp – Snail	-2.347	0.992	-2.366	**0.0858**
Shrimp – Starfish	-1.953	1.177	-1.660	0.3466
Snail – Starfish	0.394	0.992	0.397	0.9788
**Time = 2 days**				
Planarians – Shrimp	9.773	1.177	8.306	**< 0.0001**

Planarians – Snail	2.164	0.992	2.182	0.1305
Planarians – Starfish	8.810	1.177	7.487	**< 0.0001**
Shrimp – Snail	-7.608	0.992	-7.671	**< 0.0001**
Shrimp – Starfish	-0.963	1.177	-0.818	0.8459
Snail – Starfish	6.646	0.992	6.701	**< 0.0001**
**Time = 3 days**				
Planarians – Shrimp	11.455	1.177	9.735	**< 0.0001**
Planarians – Snail	2.748	0.992	2.770	0.9842
Planarians – Starfish	10.766	1.177	9.150	**< 0.0001**
Shrimp – Snail	-8.707	0.992	-8.779	**< 0.0001**
Shrimp – Starfish	-0.688	1.177	-0.585	0.1498
Snail – Starfish	8.019	0.992	8.085	**< 0.0001**
**Time = 4 days**				
Planarians – Shrimp	8.051	1.177	6.842	**< 0.0001**
Planarians – Snail	-0.356	0.992	-0.359	0.9842
Planarians – Starfish	5.559	1.177	4.725	**< 0.0001**
Shrimp – Snail	-8.407	0.992	-8.476	**< 0.0001**
Shrimp – Starfish	-2.491	1.177	-2.117	0.1498
Snail – Starfish	5.915	0.992	5.964	**< 0.0001**
**Time = 5 days**				
Planarians – Shrimp	8.784	1.177	7.465	**< 0.0001**
Planarians – Snail	1.167	0.992	1.177	0.6419
Planarians – Starfish	6.943	1.177	5.901	**< 0.0001**
Shrimp – Snail	-7.616	0.992	-7.679	**< 0.0001**
Shrimp – Starfish	-1.840	1.177	-1.564	0.4007
Snail – Starfish	5.776	0.992	5.824	**< 0.0001**
**Time = 6 days**				
Planarians – Shrimp	13.200	1.177	11.218	**< 0.0001**
Planarians – Snail	6.211	0.992	6.262	**< 0.0001**
Planarians – Starfish	9.698	1.177	8.243	**< 0.0001**
Shrimp – Snail	-6.989	0.992	-7.046	**< 0.0001**
Shrimp – Starfish	-3.501	1.177	-2.976	**0.0166**
Snail – Starfish	3.488	0.992	3.516	**0.0028**
**Time = 7 days**				
Planarians – Shrimp	2.957	1.177	2.513	0.0599
Planarians – Snail	-4.172	0.992	-4.207	**0.0002**
Planarians – Starfish	-3.376	1.177	-2.869	**0.0227**
Shrimp – Snail	-7.129	0.992	-7.188	**< 0.0001**
Shrimp – Starfish	-6.332	1.177	-5.382	**< 0.0001**
Snail – Starfish	0.797	0.992	0.803	0.8529

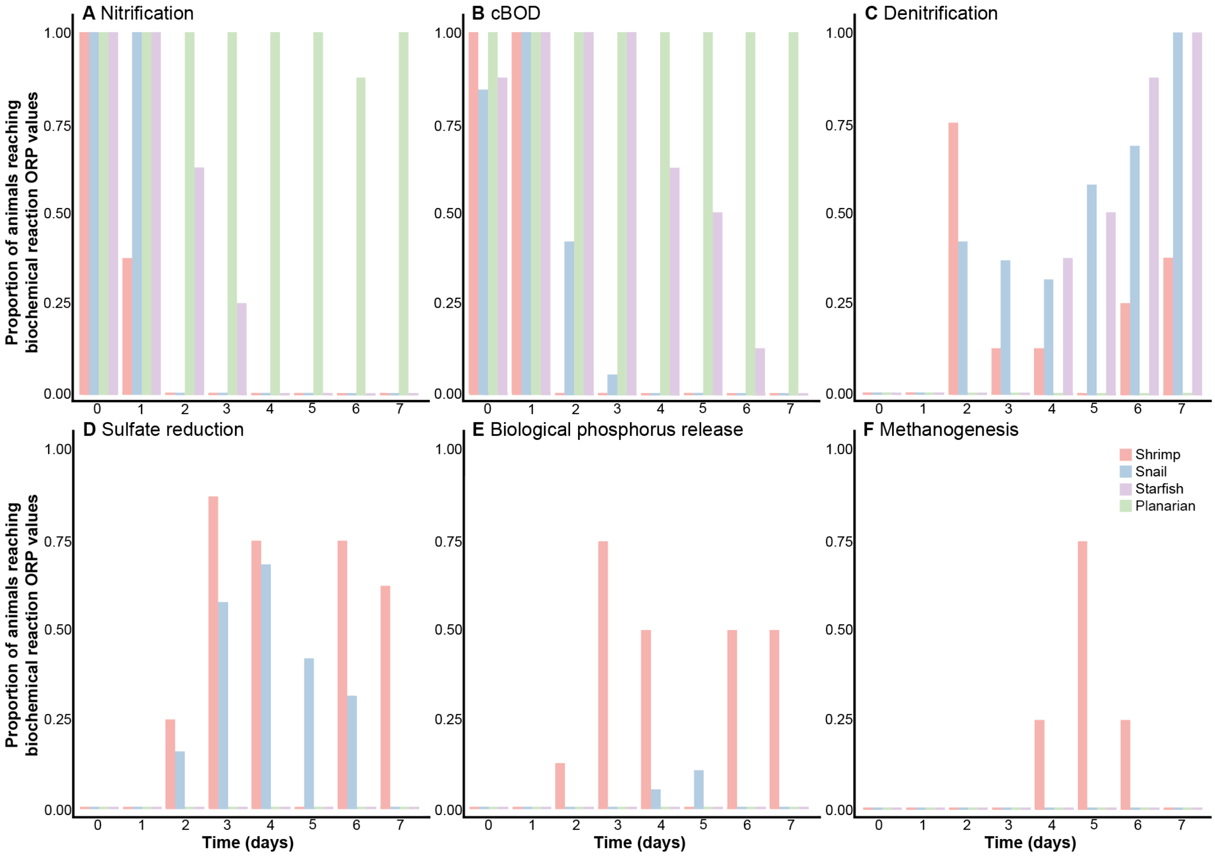

Figure S3. Proportions of animals reaching the different ORP zones over time. (A) Proportions of animals reaching nitrification, (B) carbonaceous biochemical oxygen demand (cBOD), (C) denitrification, (D) sulfate reduction, (E) biological phosphorus release, and (F) methanogenesis zones.

Table S5. Pearson correlation coefficients (*r*) comparing the values of ORP when corrected by the organic matter mass to protein:lipid, carbohydrate:lipid, and (protein + carbohydrate):lipid ratios. The Pearson correlation is two-sided.

	Degree of freedom	*r*	*t-ratio*	*p-value*
**Protein:Lipid ratio**	2	-0.9251565	-3.4468	0.07484
**Carbohydrate:Lipid ratio**	2	-0.2538133	-0.3711	0.7462
**(Protein + Carbohydrate):Lipid ratio**	2	-0.9990038	-31.66	0.0009962
