# Supplementary material for: Taxon-specific redox conditions control fossilisation pathways
Source: Nat Commun. 2025 Apr 29;16:3993. doi: 10.1038/s41467-025-59372-3 (PMC12038014; doi:10.1038/s41467-025-59372-3)
Supplement: Supplementary file 3 — Reporting Summary [file 41467_2025_59372_MOESM3_ESM.pdf]

nature portfolio

Corresponding author(s): Nora Corthésy & Farid Saleh

Last updated by author(s): 16.04.2025

Reporting Summary

Nature Portfolio wishes to improve the reproducibility of the work that we publish. This form provides structure for consistency and transparency in reporting. For further information on Nature Portfolio policies, see our Editorial Policies and the Editorial Policy Checklist.

Statistics

For all statistical analyses, confirm that the following items are present in the figure legend, table legend, main text, or Methods section.

n/a	Confirmed	
☐	☒	The exact sample size (*n*) for each experimental group/condition, given as a discrete number and unit of measurement
☐	☒	A statement on whether measurements were taken from distinct samples or whether the same sample was measured repeatedly
☐	☒	The statistical test(s) used AND whether they are one- or two-sided<br>*Only common tests should be described solely by name; describe more complex techniques in the Methods section.*
☐	☒	A description of all covariates tested
☒	☐	A description of any assumptions or corrections, such as tests of normality and adjustment for multiple comparisons
☐	☒	A full description of the statistical parameters including central tendency (e.g. means) or other basic estimates (e.g. regression coefficient) AND variation (e.g. standard deviation) or associated estimates of uncertainty (e.g. confidence intervals)
☐	☒	For null hypothesis testing, the test statistic (e.g. *F*, *t*, *r*) with confidence intervals, effect sizes, degrees of freedom and *P* value noted<br>*Give P values as exact values whenever suitable.*
☒	☐	For Bayesian analysis, information on the choice of priors and Markov chain Monte Carlo settings
☒	☐	For hierarchical and complex designs, identification of the appropriate level for tests and full reporting of outcomes
☐	☒	Estimates of effect sizes (e.g. Cohen's *d*, Pearson's *r*), indicating how they were calculated

Our web collection on statistics for biologists contains articles on many of the points above.

Software and code

Policy information about availability of computer code

Data collection	n/a
Data analysis	n/a

For manuscripts utilizing custom algorithms or software that are central to the research but not yet described in published literature, software must be made available to editors and reviewers. We strongly encourage code deposition in a community repository (e.g. GitHub). See the Nature Portfolio guidelines for submitting code & software for further information.

Data

Policy information about availability of data

All manuscripts must include a data availability statement. This statement should provide the following information, where applicable:

- Accession codes, unique identifiers, or web links for publicly available datasets
- A description of any restrictions on data availability
- For clinical datasets or third party data, please ensure that the statement adheres to our policy

Data needed to replicate the study is available in the supplementary materials and in the Source Data file.

Research involving human participants, their data, or biological material

Policy information about studies with human participants or human data. See also policy information about sex, gender (identity/presentation), and sexual orientation and race, ethnicity and racism.

Reporting on sex and gender	n/a
Reporting on race, ethnicity, or other socially relevant groupings	n/a
Population characteristics	n/a
Recruitment	n/a
Ethics oversight	n/a

Note that full information on the approval of the study protocol must also be provided in the manuscript.

Field-specific reporting

Please select the one below that is the best fit for your research. If you are not sure, read the appropriate sections before making your selection.

☐ Life sciences ☐ Behavioural & social sciences ☒ Ecological, evolutionary & environmental sciences

For a reference copy of the document with all sections, see nature.com/documents/nr-reporting-summary-flat.pdf

Ecological, evolutionary & environmental sciences study design

All studies must disclose on these points even when the disclosure is negative.

Study description	This study investigates the changes in redox potential during the decay of different phyla revealing the impact of taxon and size on tissue preservation potential.
Research sample	20 freshwater snails, 8 freshwater shrimps, 16 starfish, 8 planarians were used for this study.
Sampling strategy	Organisms were randomly sampled from the aquarium at the Insitute of Earth Science of the University of Lausanne. The sample size was not defined before because it was dependent of the available number of organisms and to reduce the number of euthanasia.
Data collection	Redox potential of the water surrounding decaying carcasses was measured with a microsensor by Nora Corthésy and Farid Saleh.
Timing and spatial scale	Data collection started on 02.05.2024 and the measures were taken every 24 hours for 7 days.
Data exclusions	No data was excluded
Reproducibility	All measurements were taken using a microsensor to measure the redox potential of the water around the decaying carcasses. All attempts to reproduce this experiment were successful.
Randomization	Investigators were collecting data randomly to avoid giving values to according to the organism group.
Blinding	Investigators were collecting data randomly to avoid giving values to according to the organism group.

Did the study involve field work? ☐ Yes ☒ No

Reporting for specific materials, systems and methods

We require information from authors about some types of materials, experimental systems and methods used in many studies. Here, indicate whether each material, system or method listed is relevant to your study. If you are not sure if a list item applies to your research, read the appropriate section before selecting a response.

Materials & experimental systems

n/a	Involved in the study
☒	☐ Antibodies
☒	☐ Eukaryotic cell lines
☒	☐ Palaeontology and archaeology
☐	☒ Animals and other organisms
☒	☐ Clinical data
☒	☐ Dual use research of concern
☒	☐ Plants

Methods

n/a	Involved in the study
☒	☐ ChIP-seq
☒	☐ Flow cytometry
☒	☐ MRI-based neuroimaging

Animals and other research organisms

Policy information about studies involving animals; ARRIVE guidelines recommended for reporting animal research, and Sex and Gender in Research

Laboratory animals	All animals were adults: Neocaridina davidi, Stenophysa marmorata, Asterina sp., Procotyla sp.
Wild animals	This is not applicable
Reporting on sex	The sex of the animals was not determined
Field-collected samples	This is not applicable
Ethics oversight	Ethical approval was not required for the organisms that were used. However, this study was done in respect of the 3Rs (Replace, Reduce, Refine).

Note that full information on the approval of the study protocol must also be provided in the manuscript.

Plants

Seed stocks	This is not applicable
Novel plant genotypes	This is not applicable
Authentication	This is not applicable
